# Transfusion medicine research in Africa: Insights from investigators in the field

**DOI:** 10.1111/vox.13407

**Published:** 2023-02-03

**Authors:** Tina S. Ipe, Quentin Eichbaum, Magdy El-Ekiaby, Shirley Owusu-Ofori, Marion Vermeulen, Tonderai Mapako, Claude Tayou Tagny, Bamory Dembele, Evan M. Bloch, Linda S. Barnes, AABB Global Transfusion Forum contributed equally

**Affiliations:** 1 Medical Division, Our Blood Institute, Oklahoma City, Oklahoma, USA; 2 Department of Pathology and Laboratory Medicine, University of Arkansas for Medical Sciences, Little Rock, Arkansas, USA; 3 Department of Pathology, Microbiology, and Immunology, Vanderbilt University Medical Center, Nashville, Tennessee, USA; 4 Blood Transfusion and Hemophilia Center, Shabrawishi Hospital, Cairo, Egypt; 5 Central Zonal Blood Center, National Blood Service Ghana, Kumasi, Ghana; 6 The South African National Blood Service, Roodepoort, South Africa; 7 Business Development Department, National Blood Service Zimbabwe, Harare, Zimbabwe; 8 Department of Hematology and Blood Transfusion, University of Yaounde, Yaounde, Cameroon; 9 National Blood Transfusion Center Laboratory and, Pharmaceutical and Biological Sciences Training and Research Unit, University Félix Houphouet-Boigny, Abidjan, Côte d’Ivoire; 10 Department of Pathology, Johns Hopkins University School of Medicine, Baltimore, Maryland, USA; 11 School of Public Health, University of Illinois, Chicago, Illinois, USA; 12 Linda S. Barnes Consulting, Seattle, Washington, USA

**Keywords:** Africa, qualitative research, transfusion medicine

## Abstract

**Background and Objectives:**

Research in low-resource settings is inherently challenging. We sought to assess the factors that have impeded or facilitated transfusion medicine (TM) research in various African settings.

**Materials and Methods:**

A qualitative case study was conducted of selected investigators in Africa; selection was based on productivity-spanning publication, leadership and research in TM. We designed a questionnaire to explore the factors impeding or facilitating TM research to understand the impact on the investigators’ careers. Written responses were independently coded and double-checked for precision. Qualitative analysis was conducted, whereby responses were grouped thematically and clustered by relationship. The initial findings were discussed with respondents to validate and refine the interpretations. The recorded transcript was analysed and incorporated into the final analysis.

**Results:**

Six investigators participated in the study. Their responses yielded 471 coded comments: 389 from the questionnaires and 82 from the ensuing discussion. The most frequently cited factors described included knowledge and intellectual abilities (*n* = 104), personal effectiveness (*n* = 99), research and governance structure (*n* = 97), and engagement, influence and impact (*n* = 75). Four relationship clusters emerged from the facilitators (*n* = 42), barriers (*n* = 28), and common approaches (*n* = 26) to research, informing summary themes of adaptation, collaboration, perseverance, and resiliency.

**Conclusion:**

Individual attributes were found to be central to a successful TM research career in African settings. However, given other public health priorities and constraints, interpersonal relationships, organizational structures and the broader research context were important to TM researchers. Overcoming complexities demands adaptation, collaboration, perseverance and resiliency.

## INTRODUCTION

Healthcare research is essential in low- and middle-income countries (LMICs) where the burden of disease is high but the resources are limited, thus underscoring the need for locally relevant, innovative, evidence-based and sustainable solutions to effect meaningful change [1, 2]. Research in low-resource settings is inherently challenging for local investigators [3]. Such is the case in large parts of Africa, where the challenges span funding, infrastructure, education and training [4, 5]. The challenges are amplified in niche disciplines, such as transfusion medicine (TM), that garner little attention.

TM researchers in Africa are grossly under-represented in the published literature. On average, 38 papers were published annually by researchers in sub-Saharan Africa (SSA) between 2008 and 2015, almost half (41%) of which focused on transfusion-transmitted infections [6]. Only a third (34%) of the publications were in transfusion-specialist journals, and an overwhelming majority of publications stemmed from collaborations outside of SSA. Consequently, policy recommendations originating in high-income countries such as those in Europe and North America are advanced where regional research might otherwise be used to guide practices that would be more appropriate and successful in a local context [7]. Examples include optimal blood donor engagement and testing strategies that increase the benefits of blood availability balanced against the risk of transfusion-transmitted infections in austere environments.

The tepid publication record may reflect a more systemic problem. Specifically, a framework for achieving success as a TM researcher in various African countries is lacking. Such a framework or roadmap could facilitate research careers for aspiring trainees and junior investigators to navigate the obstacles and leverage learnings from well-published TM researchers within the African context. The Association for the Advancement of Blood and Biotherapies (AABB) Global Transfusion Forum (GTF) Research Subcommittee undertook a qualitative study of this issue. A sample of TM experts from different regions in Africa were invited to share their perspectives to understand better the opportunities and challenges faced by African researchers. We sought to offer guidance to trainees and junior investigators, drawing on others’ first-hand regional knowledge and experience of the TM research environment in Africa.

## STUDY DESIGN AND METHODS

We employed a methodology known as interpretative phenomenology, whereby the lived experience of the research participant is incorporated into the research study in a participatory way [8]. Using a qualitative approach and two-phase case study design, the AABB GTF Research Subcommittee developed a questionnaire (Data S1) to describe the attributes, facilitators and challenges that either favoured or impeded the success of the participating investigators [8]. This allowed for the ascertainment of self-described factors that favour or impede the success of TM researchers. The questionnaire comprised 12 questions covering topics such as knowledge and intellectual ability, personal effectiveness, research governance and organization, engagement, influence and impact [3]. Responses were entered as free text.

The AABB GTF Research Subcommittee selected the invited respondents (i.e., TM research investigators). Selection of participants was based on an individual’s publication record, research funding, engagement in leadership positions in the field of TM (e.g., Africa Society for Blood Transfusion) and reputation in TM. By design, the study was limited to a few notable investigators, with the intent to represent each of the geographic regions of Africa (i.e., Central, East, North, Southern and West Africa). The investigators were invited via email to participate. The questionnaire was distributed electronically to those who agreed to participate in the study. The responses were received by email from February to December 2020.

### Data analysis

The analysis was led by a researcher experienced in qualitative methods. The respondents’ free-text responses were received as Microsoft Office 365 Word (Microsoft, Seattle, WA, USA) files and analysed using MAXQDA 2020 (VERBI Software, Berlin, Germany), a software program that is designed for qualitative and mixed-methods research. We chose to use a priori constructs as concepts compiled from an existing framework to facilitate interpretation given the contextual nature of the study while allowing for emergent concepts. The Vitae Research Development Framework (RDF) [9] was used to characterize the concepts that emerged from the responses. The RDF was created by researchers in the United Kingdom who identified factors associated with successful research careers, developed from empirical data collected through interviews. The RDF recognizes four central factors and subordinate considerations (i.e., behavioural characteristics and environmental factors) that are important for success in research (Table 1). The four prominent factors are (a) knowledge and intellectual abilities, (b) personal effectiveness, (c) research governance and organization and (d) engagement, influence and impact. In addition, there are 12 sub-constructs; definitions are provided in the Codebook (Data S2). These factors informed an a priori coding scheme and the associated definitions that were incorporated into the Codebook.

Two independent coders coded at a paragraph level, initially assigning parent codes, followed by sub-codes; the responses were reviewed independently to improve inter-rater reliability. Construct and sub-construct definitions were refined to enhance consistent understanding of the application. Where appropriate, segments were multi-coded to fully capture the meaning of the content, including intersections. Emergent codes were added throughout the coding process, informed by concurrent in-document memos.

The frequency of codes within and across the responses informed patterns. Themes were developed by summarizing the coded content within and across the questionnaire responses. These themes were clustered by proximity to understand the relationships with facilitative features and barriers described by the respondents. This approach illustrates the relative emphasis of each of the features and their relatedness (i.e., intersections). Following the initial analysis of the questionnaire responses, the preliminary findings were reviewed and discussed with two of the respondents in a recorded webinar. This member-checking approach captured additional interpretive suggestions and contextual nuance and incorporated additional insights. The recording from the discussion was transcribed, coded and analysed using the same codebook, repeating the same thematic approach to enrich and enhance the findings. Results from both sets of data (i.e., the questionnaires and the member-checking discussion) were interpreted separately and together.

### Human subjects

The study was approved by the institutional review boards (IRBs) at the University of Arkansas for Medical Sciences and Johns Hopkins University School of Medicine before initiation.

## RESULTS

Of the 10 invited researchers, 6 (60%) agreed to participate and responded to the questionnaire. All respondents described being actively engaged in TM research. The respondents were from the following countries: Cameroon, Cote d’Ivoire, Egypt, Ghana, South Africa and Zimbabwe. Three respondents were physicians (MBchB/MBBch/MD) having between 10 and 30 years of experience in TM research. Two respondents held the PhD degree with more than 15 years of experience in TM-related fields. One respondent is a PhD candidate with more than 20 years of experience in senior technical/management roles including TM research. All reported receiving grant funding and holding a considerable record of peer-reviewed literature in TM (median 35.5; range 6–57).

### Questionnaire responses

**FIGURE 1:**
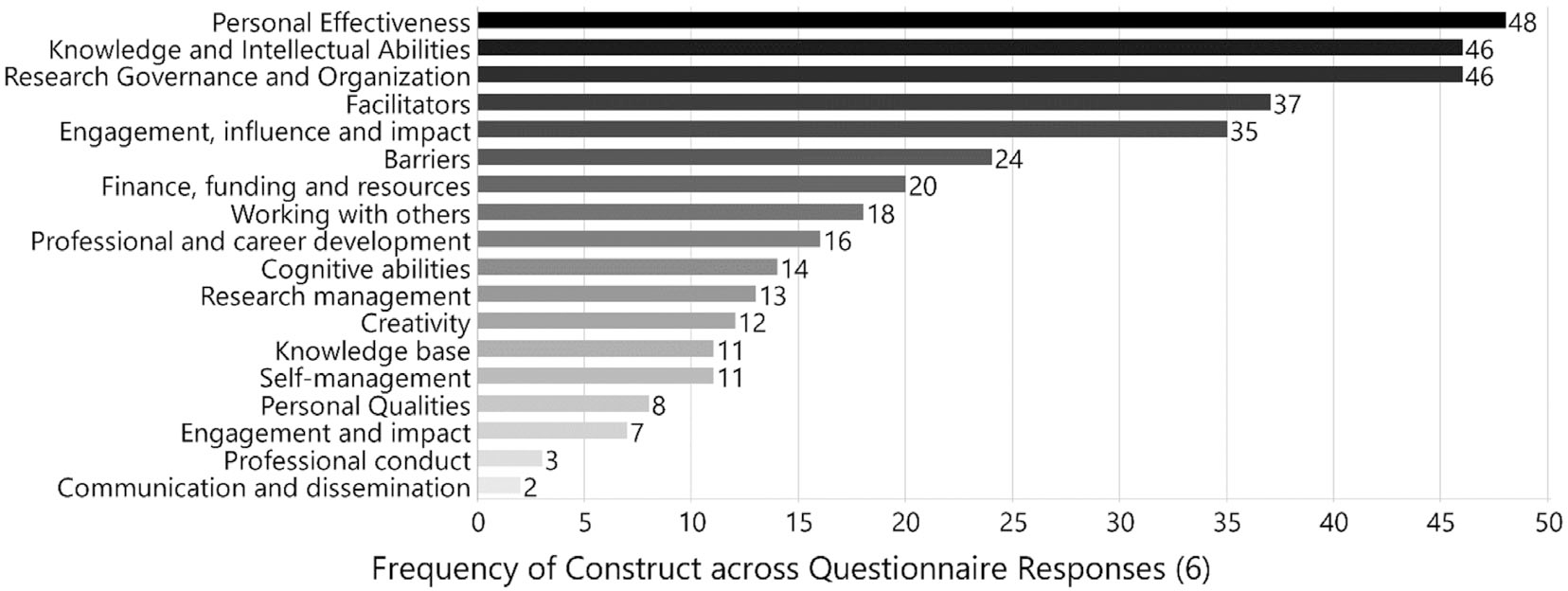
Code frequency by construct across the accumulated questionnaires received from African transfusion medicine researchers.

A total of 389 codes were applied to written segments, covering 94% (range 89%–96%) of the content of the questionnaires. The two coders reached a significant inter-coder agreement (91%) through iterative comparisons and improvement of definitions. Across all questionnaire responses (Figure 1), the most frequently referenced factors by the number of coded segments, in parentheses, were personal effectiveness (*n* = 48; 12.3%) followed by knowledge and intellectual abilities (*n* = 46; 11.8%) and research governance and organization (*n* = 46; 11.8%). More facilitators (*n* = 37; 9.5%) were described than barriers (*n* = 24; 6.2%). The least commonly mentioned considerations included personal qualities (*n* = 8; 2.1%), engagement and impact (*n* = 7; 1.8%), professional conduct (*n* = 3; 0.8%), and communication and dissemination (*n* = 2; 0.5%).

### Member-checking discussion

A total of 82 segments were coded from the discussion with respondents. The most frequently cited factor was knowledge and intellectual abilities (*n* = 10; 12.2%), followed by personal effectiveness (*n* = 8; 9.8%) and research governance organization (*n* = 8; 9.8%). Creativity (*n* = 7; 8.5%) and engagement, influence and impact (*n* = 7; 8.5%) were also rated high. We observed that personal qualities (*n* = 2; 2.4%) and professional conduct (*n* = 1; 1.2%) were less frequently mentioned.

### Combined data

**FIGURE 2:**
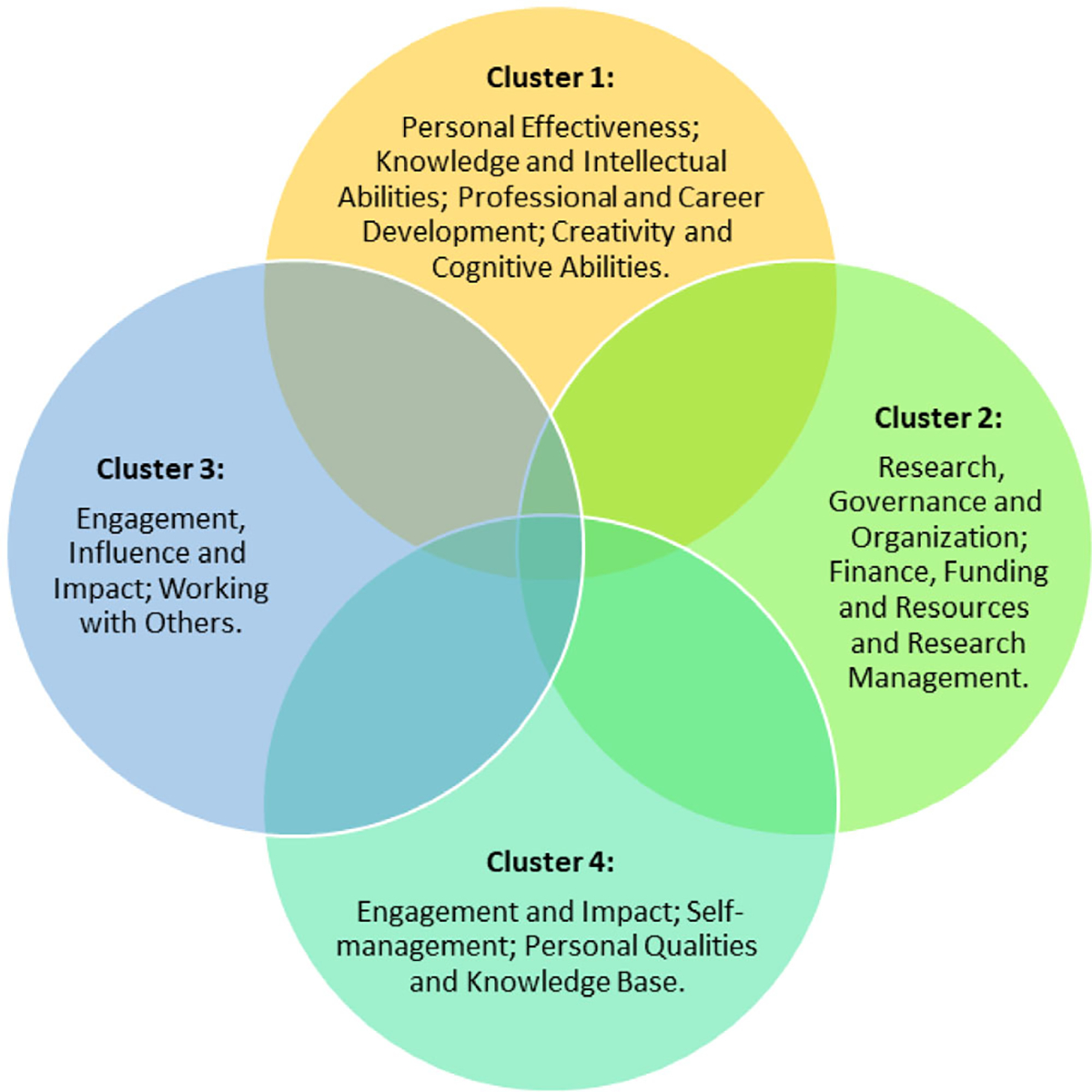
Four clusters that were developed based on the intersection of concepts after combining responses from the questionnaires and the member-checking discussion.

Across the collective questionnaire responses and member-checking discussion, we observed code intersections occurring when a response reflected more than one construct, leading to multi-coding. These intersections and co-occurrences were also examined. The most frequent intersections occurred between personal effectiveness and knowledge and intellectual abilities (*n* = 31 co-occurrences). We also observed that facilitators co-occurred with research governance and organization (*n* = 23), personal effectiveness (*n* = 20) and knowledge and intellectual abilities (*n* = 20). However, we noted that research and governance were most frequently identified as the highest ranking barriers (*n* = 18), followed by engagement, influence and impact (*n* = 13). Using the code map intersection, we found four relationships, herein called clusters, by map position between pairs of codes (Figure 2).

**FIGURE 3:**
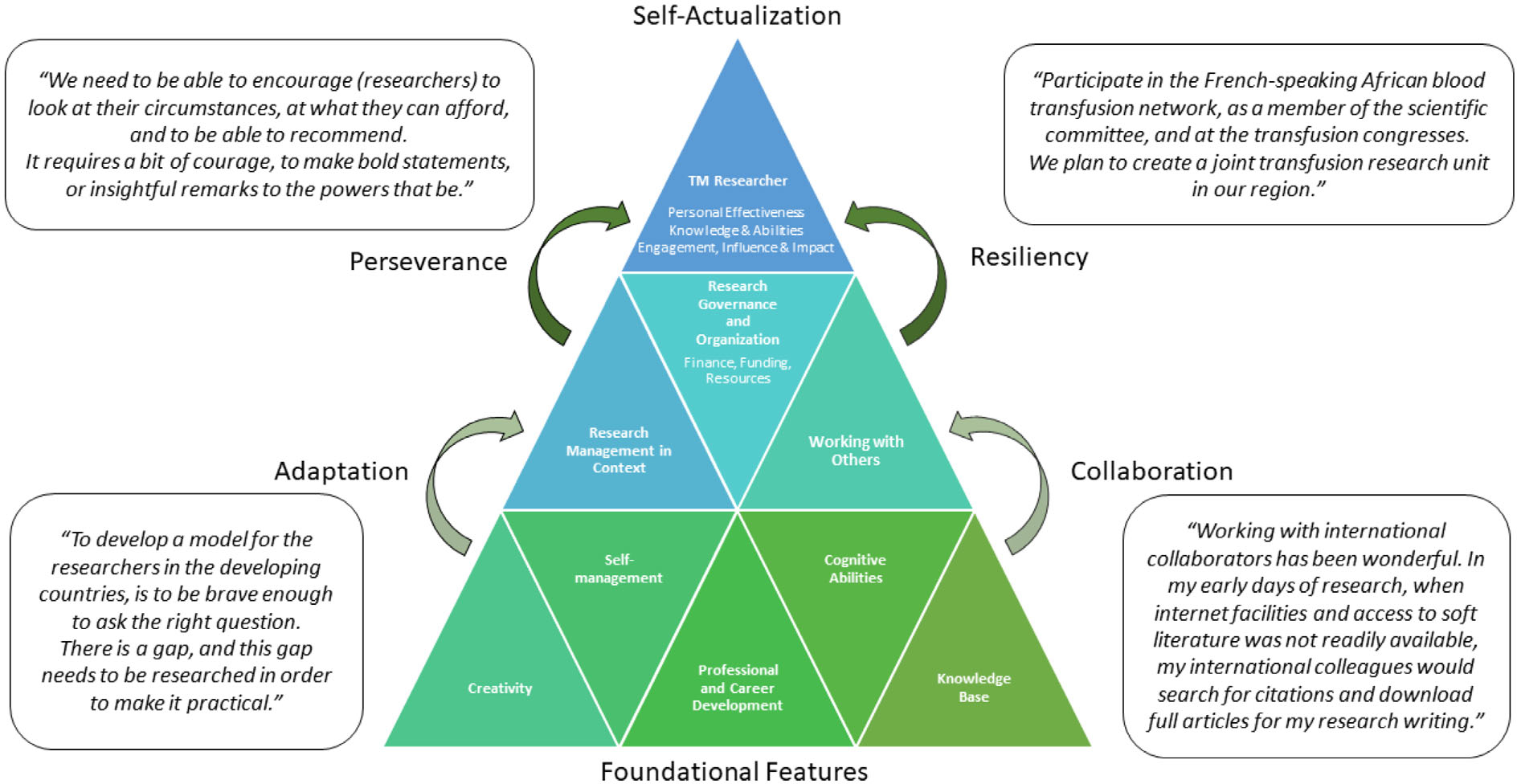
Developing Research Framework, synthesized from the four clusters illustrating the how foundational individual attributes build towards interpersonal and contextualized approaches, leveraging work with others and available financial resources, through personal effectiveness, knowledge and abilities and engagement, influence and impact. Common themes for overcoming hurdles included adaptation, collaboration, perseverance and resiliency.

The clusters were further examined to identify the common developmental approaches described by the researchers as they advanced in their TM careers. Characterized as a Developing Researcher Framework, Figure 3 captures key attributes that arose from the study to facilitate advancement and overcome barriers to achieve a successful career as a TM researcher. Derived from notable quotes in the words of the researchers, these approaches are summarized as an adaptation of the research agenda to make it practicable in low-resource settings: international collaborations with other TM researchers and resiliency to overcome barriers through joint efforts to achieve the intended impact. We mapped the relationships of the themes to a hierarchy from foundational features to the self-actualization of the successful TM researcher. A set of recommendations compiled from the researchers summarizes the advice given to those pursuing a TM research career in the African context (Table 2).

## DISCUSSION

Our study findings highlight themes from the self-described perspectives of TM researchers who have had successful TM research careers in parts of Africa. Key findings emphasize personal effectiveness combined with knowledge and intellectual abilities. Research governance and organization, including funding, had a significant impact on individuals’ careers, favourable or otherwise. While not unique to an African setting, adaptation, collaboration, perseverance and resiliency notably contributed to positive outcomes. There was a perceived need for local, innovative, evidence-based and sustainable solutions, which have already been identified as deficient in the context of TM research in Africa and other low-resource settings [4–6]. Additionally, the respondents’ lesser emphasis on communication and dissemination was striking, potentially explaining under-representation in scientific publication [2, 7].

A qualitative approach was better equipped to understand the developmental trajectories of a sampling of research investigators in Africa. By drawing on the researchers’ perspectives, a qualitative approach affords a depth of context and insight, which is frequently lacking in quantitative methods. Our study found intersecting patterns in the questionnaire responses and discussion content supporting a strong relationship of a researcher’s knowledge and abilities combined with personal effectiveness; these were key factors in facilitating the researchers’ successful career development. Personal effectiveness, which encompassed unique personal qualities, self-management and professional and career development, was the dominant quality attributed to successful TM researchers in Africa. In other words, the personal initiative to conduct and sustain their research goals was vital to the researcher’s success. To develop their professional TM research career, respondents shared that self-management was essential to creating a robust knowledge base and cognitive abilities as a researcher. Several respondents described the lack of training infrastructure in their countries to learn research skills as an impediment. Attendance in conferences, review courses or post-graduate education (e.g., in the United States and Europe) was essential to obtain and maintain valuable skills in research methodology and manuscript writing. There are successful examples, albeit few, where courses have been devised to impart foundational skills in either clinical TM or related research [3, 10–13].

Self-discipline was key to generating manuscripts and publications, where research tasks were often undertaken outside work hours. The researchers were relentless in their research pursuits, some describing self-funding of their research. Creativity was apparent, with one respondent sharing how they acted on the opportunity to become a ‘research officer’, which enabled them to further develop their research expertise as part of a formal professional role. However, another respondent succinctly stated, ‘You will need to have perseverance and manage your time to be able to be successful in this area. You need to want to do it’.

The respondents observed that their personal attributes and mastery alone were insufficient to ensure a successful research career in TM. These researchers described being effective because they forged collaborations with industry sponsors, in-country and international TM clinicians and other researchers. They relied on mentors to help them network and establish relationships with other TM researchers. They also encouraged their research staff to advance their research through participating in local, national and international TM research. Notably, the most impactful research topics were locally and/or regionally relevant, often informed by challenges specific to the population and settings where they worked [14–19].

Even when motivated and collaborating on important works, the researchers described being sometimes constrained by their respective regions’ research, governance and organizational structures. For example, their local environments were not conducive to research because of poor access to technology such as the Internet, lack of adequately trained research staff and the lack of internal (state, governmental or institutional) funding. The lack of administrative and technology support sometimes impaired their ability to coordinate research projects or even meet sponsors’ deadlines for those projects. This hampered their ability to engage with or influence the national and international TM community. However, the respondents described adaptive ways to move beyond these challenges through collaboration and engagement with professional societies.

Surprisingly, the respondents did not refer to the regulatory review process as a notable obstacle to research. This was not stated explicitly; rather, the omission was conspicuous. Communication and the ability to disseminate research findings were not mentioned explicitly as research and career obstacles. Of note, none of the researchers spoke about the professional conduct of their colleagues or their own as being either a facilitator or barrier to conducting research.

During the member-checking discussion, the investigators emphasized self-management as a critical characteristic of a successful individual in LMICs. However, the foundational features of the Developing Research Framework (Figure 3) built a pathway towards self-actualization built upon the innovative approaches described by accomplished TM researchers [20]. These approaches included the adaptation of research specific to the local context, collaborations with local and international researchers, perseverance marked by courage and persistence to communicate unpopular findings and resiliency to overcome barriers to research. This approach allowed them to address locally pertinent research questions having a more significant bearing on policymakers, administrators, clinicians and public health collaborations. Examples of local research capacity building in Africa include the Francophone Africa Transfusion Medicine Research Training network, T-REC, and the NIH REDS-III and Fogarty South Africa progammes [13]. These research programmes have enrolled trainees at all levels, from short-term course participants in epidemiology to Masters and PhD candidates. It has increased the number of TM research publications originating from Africa, with at least 60 new manuscripts in the past several years. These programmes noted shortages of mentors and grant-writing skills as challenges faced by trainees. While these contextually rich insights are poorly described in the peer-reviewed literature, they may support advancing TM researchers in the African context and beyond.

This study has several limitations. First, the small sample size (i.e., small number of participants and skewed geographic representation) warrants highlighting. Although our sampling plan was broad, not all African regions were represented. However, this was deemed sufficient for a qualitative analysis given the narrow scope of the study and thematic saturation. We acknowledge that our definitions of ‘success’ and ‘productivity’ in research, as defined by publications, contributions to professional societies and grant funding, may not be the only measures of a successful career or impactful contribution. We did not solicit opinions of those who attempted a TM research career but did not meet our definitions. While such information may serve as an important comparison, this was not the focus of our study. We appreciate that the respondents’ experience varied by individual and their context, representing a limitation in transferability to other settings. We applied reflexivity and member-checking to validate the observed patterns and found the thematic similarities striking. Although this study focused on experiences in African settings, its findings may be relevant to TM researchers with similar backgrounds and other comparable LMIC contexts.

The findings of this study point to the remarkable ability of a subset of African TM investigators to navigate a range of impediments and obstacles in conducting research in Africa. These accomplished TM researchers exercised personal effectiveness combined with knowledge and abilities. Their successes were further enhanced through adaptation, collaboration, perseverance and resiliency. A pipeline of future researchers is critical to increase the capacity of research in LMICs, enticing aspiring trainees. These findings warrant further exploration, mainly to understand how to teach, mentor and expand TM research specific to Africa.

## Supplementary Material

Data S2

Data S1

## Figures and Tables

**TABLE 1:** A priori constructs, sub-constructs, and definitions applied to code questionnaires and the subsequent member-checking discussion based on the Vitae Researcher Development Framework (Vitae, © 2010 Careers Research and Advisory Centre [CRAC] Limited) [5].

Construct (concept)	Sub-construct (factors)	Definition
Personal effectiveness	Personal qualities Self-management Professional and career development	Personal qualities and approach to be an effective researcher
Knowledge and intellectual abilities	Knowledge base Cognitive abilities Creativity	Knowledge, intellectual abilities and techniques to do the research
Research governance and organization	Professional conduct Research management Finance, funding and resources	Knowledge of the standards and requirements, and professionalism to do the research
Engagement, influence and impact	Working with others Communication and dissemination Engagement and impact	Knowledge and skills to work with others and ensure wider impact of research

**TABLE 2:** Consolidated recommendations for future transfusion medicine (TM) researchers, compiled from the questionnaire responses and discussion.

• Define your research interest in TM.
∘ Always start your research by asking a question.
∘ Perform a thorough literature review.
∘ Define your aim of research.
∘ Study options for funding your research.
∘ Get trained on how to write a proper manuscript to publish the results of your research.
• Consider research as a career. There will be initial hurdles and disappointments, but there are high rewards to be gained if efforts are sustained quite well.
∘ Identify mentors in your line of research interest.
∘ Pursue publication of your research work.
∘ Advance your education and embark on operational research.
∘ Develop relevant skills for research. Aim to embark on ethical conduct of research always.
∘ Utilize available free educational opportunities.
∘ Endeavour to attend to scientific congresses as an active participant (present some scientific work).
∘ Join allied associations such as Africa Society for Blood Transfusion (AfSBT), International Society of Blood Transfusion (ISBT), Association for the Advancement of Blood and Biotherapies (AABB), etc.
• Explore research networking opportunities in Africa Society for Blood Transfusion, International Society for Blood Transfusion and the Association for the Advancement of Blood and Biotherapies to enhance research interests and collaborations with other TM researchers.

## Highlights

- Transfusion medicine researchers, both clinical and non-clinical, in Africa do not publish as frequently as their counterparts in other continents.
- This study showed personal effectiveness, knowledge and intellectual capability as attributes of successful researchers in Africa.
- Creating a pipeline of researchers in Africa is vital to building research capacity, not only in this continent but also in other low- and middle-income countries.

## References

[1] Alemayehu C, Mitchell G, Nikles J. Barriers for conducting clinical trials in developing countries- a systematic review. Int J Equity Health. 2018;17:37. 29566721 10.1186/s12939-018-0748-6 PMC5863824

[2] Custer B, Zou S, Glynn SA, Makani J, Tayou Tagny C, El Ekiaby M, Addressing gaps in international blood availability and transfusion safety in low- and middle-income countries: a NHLBI workshop. Transfusion. 2018;58:1307–17. 29542130 10.1111/trf.14598 PMC6510980

[3] Murphy EL, McFarland W, Lefrère J-J. Teaching transfusion medicine research methods in the developing world. Transfusion. 2009;49:1532–4. 19732403 10.1111/j.1537-2995.2009.02299.x PMC2740979

[4] Weimer A, Tagny CT, Tapko JB, Gouws C, Tobian AAR, Ness PM, Blood transfusion safety in sub-Saharan Africa: a literature review of changes and challenges in the 21st century. Transfusion. 2019;59:412–27. 30615810 10.1111/trf.14949

[5] Ifland L, Bloch EM, Pitman JP. Funding blood safety in the 21st century. Transfusion. 2018;58:105–12. 29030857 10.1111/trf.14374

[6] Fisher A, Wallis S, Hassall O, Martin R, Bates I. Collaborations on blood transfusion research in sub-Saharan Africa: who, what and where. Vox Sang. 2020;115:221–32. 32026497 10.1111/vox.12884 PMC7187137

[7] Bates I Final Report Summary - T-REC (Building research capacity of blood transfusion services in Africa) [monograph on the internet]. 2015 [cited 2022 Aug 10]. Available from: https://cordis.europa.eu/project/id/266194/reporting.

[8] Yin RK. Case study research and applications: design and methods. 6th ed. United States: SAGE Publications; 2017.

[9] Vitae. Vitae RDF [monograph on the internet]. 2021 [cited 2022 Aug 10]. Available from: https://www.vitae.ac.uk/images/researchers-professional-development/TransparentRDFgraphic.png/view.

[10] Sibinga CT. Filling a gap in transfusion medicine education and research. Transfus Med Rev. 2009;23:284–91. 19765517 10.1016/j.tmrv.2009.06.003

[11] Guarner J, Amukele T, Mehari M, Gemechu T, Woldeamanuel Y, Winkler AM, Building capacity in laboratory medicine in Africa by increasing physician involvement: a laboratory medicine course for clinicians. Am J Clin Pathol. 2015;143:405–11. 25696799 10.1309/AJCPNYT1WPSRCLC6

[12] Louw VJ, Nel MM, Hay JF. Postgraduate education in transfusion medicine in the absence of formal residency training: assessment of factors needed to develop and sustain a postgraduate diploma program. Transfus Apher Sci. 2013;49:681–6. 22868186 10.1016/j.transci.2012.07.003

[13] Mapako T, Tagny CT, Laperche S, Bates I, Murphy EL. Building capacities in research for blood services in Africa. Transfus Clin Biol. 2021;28:171–4. 33516885 10.1016/j.tracli.2021.01.006

[14] Tagny CT, Diarra A, Yahaya R, Hakizimana M, Nguessan A, Mbensa G, Characteristics of blood donors and donated blood in sub-Saharan Francophone Africa. Transfusion. 2009;49:1592–9. 19389036 10.1111/j.1537-2995.2009.02137.x

[15] Vermeulen M, Swanevelder R, Chowdhury D, Ingram C, Reddy R, Bloch EM, Use of blood donor screening to monitor prevalence of HIV and hepatitis B and C viruses, South Africa. Emerg Infect Dis. 2017;23:1560–3. 28820374 10.3201/eid2309.161594 PMC5572879

[16] Mapako T, Mvere DA, Chitiyo ME, Rusakaniko S, Postma MJ, van Hulst M. Human immunodeficiency virus prevalence, incidence, and residual transmission risk in first-time and repeat blood donations in Zimbabwe: implications on blood safety. Transfusion. 2013; 53:2413–21. 23789991 10.1111/trf.12311

[17] Owusu-Ofori S, Temple J, Sarkodie F, Anokwa M, Candotti D, Allain J-P. Predonation screening of blood donors with rapid tests: implementation and efficacy of a novel approach to blood safety in resource-poor settings. Transfusion. 2005;45:133–40. 15660820 10.1111/j.1537-2995.2004.04279.x

[18] Ala F, Allain JP, Bates I, Boukef K, Boulton F, Brandful J, External financial aid to blood transfusion services in sub-Saharan Africa: a need for reflection. PLoS Med. 2012;9:e1001309. 22984355 10.1371/journal.pmed.1001309 PMC3439367

[19] Kouao MD, Dembele B, N’Goran LK, Konate S, Bloch E, Murphy EL, Reasons for blood donation deferral in sub-Saharan Africa: experience in Ivory Coast. Transfusion. 2012;52:1602–6. 22780941 10.1111/j.1537-2995.2012.03756.x PMC3658824

[20] Kaufman S What does it mean to be self-actualized in the 21st century? Sci Am. 2018. https://blogs.scientificamerican.com/beautiful-minds/what-does-it-mean-to-be-self-actualized-in-the-21st-century/. Accessed December 12, 2021.

