# Supplementary material for: Transfusion medicine research in Africa: Insights from investigators in the field
Source: Vox Sang. Author manuscript; Available in PMC 2024 Apr 10. (PMC11005390; doi:10.1111/vox.13407)
Supplement: Data S2 [file NIHMS1978309-supplement-Data_S2.docx]

**Codebook**


African Research.mx20


11/5/2021

**Code System**

| Knowledge and Intellectual Abilities |
| --- |
| Knowledge base |
| Cognitive abilities |
| Creativity |
| Personal Effectiveness |
| Personal Qualities |
| Self-management |
| Professional and career development |
| Research Governance and Organization |
| Professional conduct |
| Research management |
| Finance, funding, and resources |
| Engagement, influence and impact |
| Working with others |
| Communication and dissemination |
| Engagement and impact |
| Facilitators |
| Barriers |

**Knowledge and Intellectual Abilities**

The knowled, intellectual abilities and techniques to do research

**Knowledge base**

education (undergrad, grad etc)

**Cognitive abilities**

research questions

May also include pursuit of grants, post-grad education, specialization in discipline

**Creativity**

research questions and overcoming research obstacles, innovation, new contributions to the field.

**Personal Effectiveness**

The personal qualities and approach to be an effective researcher

**Personal Qualities**

innate drive as a researcher

**Self-management**

problem solving, working with others, initiative

included effectiveness in managing change/research agendas

**Professional and career development**

how they advanced their career

**Research Governance and Organization**

The knowledge of the standards, requirements, and professionalism to do research,

ability to apply for funding or acquire funding

**Professional conduct**

manuscripts, successes as a researcher, advancement in rank, mentees

**Research management**

how they managed research resources

also included organizing around researching regional problems

**Finance, funding, and resources**

grants awarded, sponsorships, role in awards (e.g., PI, sub-PI, collaborator)

**Engagement, influence and impact**

The knowledge and skills to work with others and ensure the wider impact of research

**Working with others**

examples of collaborations, multi-disciplinary projects, co-authorship

**Communication and dissemination**

how findings led to public dissemination, publication, implementation, new research projects

**Engagement and impact**

how they networked, level of application of research findinges (e.g., hi-index journals, new policies, standards, approaches)

**Facilitators**

factors that enabled competencies, accomplishments, impact

**Barriers**

factors that presented roadblocks, interferred with development of competencies, achievement of accomplishments, minimized impact.
