# Supplementary material for: Transfusion medicine research in Africa: Insights from investigators in the field
Source: Vox Sang. Author manuscript; Available in PMC 2024 Apr 10. (PMC11005390; doi:10.1111/vox.13407)
Supplement: Data S1 [file NIHMS1978309-supplement-Data_S1.docx]

**AABB Global Transfusion Forum**

**TRANSFUSION MEDICINE RESEARCH IN AFRICA: INSIGHTS FROM INVESTIGATORS IN THE FIELD**

***A Qualitative Study to determine resources and challenges pertaining to***

***Transfusion Medicine research in Africa***

**Questions included in the Questionnaire**

1. Draft a brief description of your
   1. academic training:
   2. current position:
   3. major research focus:
   4. number of years working in TM research: (comment on how you developed your research interests)
2. Briefly describe the research infrastructure at your institution
   1. Name of primary institution where you currently work:
   2. How many faculty/personnel are engaged in TM research in your institution?
   3. Approximately how many projects were active in the last year?
   4. How were those projects funded? (list all that apply)
      1. International/governmental support
      2. National/governmental support
      3. Institutional grant
      4. Private donor
3. What were the opportunities that led to your success/successes in research?
4. What do you perceive to be the major challenges/barriers to research in your region (note region)? Break down by
   1. Historical-
   2. Current- and
   3. Future/anticipated obstacles
5. Please describe ways in which you have been able to overcome the identified obstacles (provide direct examples):
6. How does one obtain research experience and training, in your region (specifically in transfusion medicine?
7. What are the major research gaps in transfusion medicine in your region:
8. For human subjects’ research, what resources are available to ensure ethical practices?
9. What resources are needed in your region to further research in Transfusion Medicine:
10. Describe initiatives, past, underway, or planned to promote research in your region:
11. Provide examples of initiatives that have failed or proved to be suboptimal:
12. Offer advice for junior investigators embarking in a career in Transfusion Medicine in your region:
